# Pharmacokinetic model of unfractionated heparin during and after cardiopulmonary bypass in cardiac surgery

**DOI:** 10.1186/s12967-015-0404-5

**Published:** 2015-02-01

**Authors:** Zaishen Jia, Ganzhong Tian, Yupeng Ren, Zhiquan Sun, Wei Lu, Xiaotong Hou

**Affiliations:** Department of Extracorporeal Circulation, Center for Cardiac Intensive Care, Beijing Anzhen Hospital, Capital Medical University, Beijing Institute of Heart Lung and Blood Vessel Disease, No. 2 Anzhen Road, Chaoyang District, Beijing, 100029 China; Department of Pharmaceutics, School of Pharmaceutical Science, Peking University Health Science Centre, No.38 Xueyuan Road, Haidian District, Beijing, 100191 China

**Keywords:** Cardiopulmonary bypass, Cardiac surgery, Pharmacokinetic model, Unfractionated heparin

## Abstract

**Background:**

Unfractionated heparin (UFH) is widely used as a reversible anti-coagulant in cardiopulmonary bypass (CPB). However, the pharmacokinetic characteristics of UFH in CPB surgeries remain unknown because of the lack of means to directly determine plasma UFH concentrations. The aim of this study was to establish a pharmacokinetic model to predict plasma UFH concentrations at the end of CPB for optimal neutralization with protamine sulfate.

**Methods:**

Forty-one patients undergoing CPB during cardiac surgery were enrolled in this observational clinical study of UFH pharmacokinetics. Patients received intravenous injections of UFH, and plasma anti-F_IIa_ activity was measured with commercial anti-F_IIa_ assay kits. A population pharmacokinetic model was established by using nonlinear mixed-effects modeling (NONMEM) software and validated by visual predictive check and Bootstrap analyses. Estimated parameters in the final model were used to simulate additional protamine administration after cardiac surgery in order to eliminate heparin rebound. Plans for postoperative protamine intravenous injections and infusions were quantitatively compared and evaluated during the simulation.

**Results:**

A two-compartment pharmacokinetic model with first-order elimination provided the best fit. Subsequent simulation of postoperative protamine administration suggested that a lower-dose protamine infusion over 24 h may provide better elimination and prevent heparin rebound than bolus injection and other infusion regimens that have higher infusion rates and shorter duration.

**Conclusion:**

A two-compartment model accurately reflects the pharmacokinetics of UFH in Chinese patients during CPB and can be used to explain postoperative heparin rebound after protamine neutralization. Simulations suggest a 24-h protamine infusion is more effective for heparin rebound prevention than a 6-h protamine infusion.

## Background

Unfractionated heparin (UFH) is an anionic mixture of highly sulfated linear glucosamine-glycans with varying molecular weights (3–30 kD) [1,2]. The anti-coagulation effect of heparin is dependent upon binding with the serine protease inhibitor anti-thrombin III (ATIII) [3]. Binding with ATIII increases the inhibitory activity of ATIII against both thrombin (F_IIa_) and factor Xa (F_Xa_) and other serine proteases in the coagulation cascade by over 1000-fold [2,4]. Human F_IIa_ is much more sensitive than F_Xa_ to inhibition mediated by the heparin-ATIII complex, so the anti-F_IIa_ assay may have a smaller lower limit of quantitation (LLOQ) [5]. We measured anti-F_IIa_ activity as an index to quantify plasma UFH levels in humans [6].

The UFH half-life is 1–2 h in human plasma, depending on dose [2], as higher doses produce a prolonged half-life due to the mechanism of plasma clearance, which involves rapid distribution via UFH binding to plasma proteins and receptors on endothelial cells and macrophages, followed by slower elimination through the kidneys [5]. Thus, UFH pharmacokinetics may include a “peripheral process” by which the UFH molecule is converted from the free to the bound state. Moreover, plasma UFH concentrations may exhibit larger inter-individual variability than other anti-coagulation drugs.

UFH is widely utilized in cardiac surgery to achieve adequate anti-coagulation and to restore normal hemostasis during and after CPB. UFH anti-coagulation can be reversed through formation of a stable complex with the highly cationic protamine sulfate, although its use as a ‘neutralizer’ of UFH carries some risk. Rapid administration of protamine sulfate can cause life-threatening hemodynamic disturbances such as systemic arterial hypotension and pulmonary hypertension, along with histamine release and hypoxemia, especially at the end of CPB when the myocardium is recovering from ischemic insult [2,7,8]. Complete reversal of UFH anti-coagulation is typically achieved with an excessive dose of protamine sulfate, although this has been associated with increased bleeding and inhibition of platelet glycoprotein Ib-von Willebrand factor, increased expression of P-selectin, blockade of calcium-release channels, and negative inotropic effects [2,9]. Accurate protamine dosing requires an understanding of real plasma UFH levels. However, the pharmacokinetic profile of UFH is unknown for Asian patients undergoing CPB.

Since 1962, Hyun and other researchers have used the phrase “heparin rebound” to describe the reappearance of UHF in circulating blood even after a dose of excess protamine sulfate [10,11]. Teoh et al. postulated that heparin rebound could be due to a portion of UFH administered during surgery that remains protein-bound and does not form a complex with protamine sulfate; these complexes dissociate slowly to produce anticoagulant effects [12]. No quantitative studies of this phenomenon have been published.

The objective of this study was to quantitate the pharmacokinetic characteristics of UFH in Chinese patients undergoing CPB, characterize the correlation between heparin rebound and UFH pharmacokinetics, and establish a pharmacokinetic model. The model can be used to simulate and optimize protamine sulfate dosing in order to minimize the side effects of protamine sulfate and restore normal hemostasis with minimal post-operative bleeding and blood transfusion.

## Methods

### Patients

After subjects with dysfunctions of the kidney, liver, or blood coagulation were excluded, 41 study patients underwent CPB during cardiac surgery (Table 1). The study protocol was approved by the Ethics Committee of Capital Medical University (Beijing, China). All subjects provided written informed consent prior to enrollment.

**Table 1:** **Baseline characteristics of patients**

**Characteristic**	**Unit**	**Number or mean (range)**
Number of patients	/	41
Body weight	kg	66 (41–82)
Sex	/	Females: 19 Males: 13
Age	Years	53.4 (18–74)
CPB time	Hours	2.04 (0.95–3.29)
Dose of UFH in priming fluid before CPB	IU	8516 (6250–10000)
First dose of UFH	IU	24805 (18750–31250)
Total dose of UFH	IU	34023 (25000–45000)

### Experimental design

Subjects had received an initial intra-venous injection of UFH (Changzhou Qianhong Biopharma, Changzhou, China) at 375 IU/kg (3 mg/kg; 1 mg = 125 IU) before CPB. Ten minutes after the initial dose, the first blood sample was collected from the jugular vein. The CPB pipelines were primed with 1500 mL of balanced solution and a second UFH dose (1 mg/kg). The CPB flow was maintained at 2.2–2.4 L/min/m^2^ with gravity siphon venous drainage. The temperature of the CPB system drifted to 32°C. The targeted mean perfusion pressure was 50–70 mmHg. Myocardial preservation was achieved with blood cardioplegic solution; ultrafiltration was avoided throughout the operation. A series of irregular UFH intravenous injections were administered, depending on each patient’s blood coagulation activity during CPB.

Before UFH neutralization with protamine sulfate, sampling was performed at 30-min intervals. All samples were collected in 3-mL vacuum tubes buffered with sodium citrate and stored immediately at 4°C. Stored blood samples were centrifuged (2000 × *g*, 15 min) within 24 h of collection to remove platelets and hemocytes. Platelet-poor plasma (PPP) was then collected and stored at −80°C. The actual UFH dosing time and dosages of were recorded carefully.

After UFH neutralization, the sampling schedule was 2, 4, 8, 12, and 24 h. Actual sampling times deviated slightly from the schedule, so only the actual sampling times were documented.

### Sample dilution and determination of anti-F_IIa_ activity

The actual anti-F_IIa_ activity in plasma during CPB is much larger than the measurable range (0.0–0.6 IU/mL for the anti-F_IIa_ assay). Thus, samples collected before protamine neutralization were diluted with Normal Pooled Platelet-poor plasma (NPPPP) at 1:29. NPPPP was derived from PPP collected from 20 randomly selected healthy adults. Twenty parts of plasma were centrifuged separately (2000 x *g*, 15 min, at room temperature) to remove platelets, then were mixed into a single-part NPPPP.

Anti-F_IIa_ activities in plasma were determined as reported by Falkon et al. [13,14]. All assays were performed on an ACL-TOP automated coagulation assay platform (Instrumentation Laboratory, Orangeburg, NY). The Heparin Chromogenic Activity Kit 820 (American Diagnostica, Stamford, CT) was used for the anti-F_IIa_ assay.

### Modeling strategy

NONMEM (version VII, level 2.0; Icon Development Solutions, Hanover, MD) was used to establish the pharmacokinetic model, with gFORTRAN (version 4.0) as the FORTRAN compiler and platform R (version 2.15.0) as the statistical and plotting software. All modeling and simulation procedures were performed on an operative platform for NONMEM known as “Perl speaks NONMEM” (PsN; version 3.4.0). First-order conditional estimation (FOCE) was the chosen algorithm. Results with p < 0.01 were considered significant.

**Figure 1:**
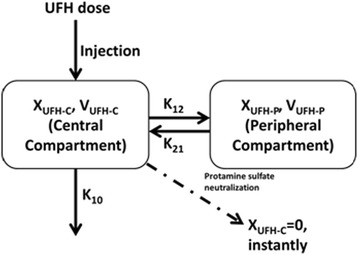
**Two-compartment model with intravenous injection and first-order elimination.** *X*_*UFH-C*_ and *X*_*UFH-P*_ represent the amount of UFH in central and peripheral compartments respectively; *V*_*UFH-C*_ and *V*_*UFH-P*_ are the apparent volumes of distribution for the central and peripheral compartments respectively.

Our data involved a series of post-neutralization points indicating heparin rebound, which cannot be explained by a mono-compartment model. Thus, the base model that provided the best data fit was a two-compartment model with first-order elimination and multiple irregular intravenous administration of UFH (Figure 1).

The base model was a typical two-compartment model with multiple dosing during surgery. However, due to injection of excessive protamine sulfate, the amount of UFH in the central compartment was supposed to be instantly cleared. Thus, *X*_*UFH-C*_ was set to 0 at that time, whereas the amount of UFH in the peripheral compartment remained unaffected. Distribution to the central compartment followed the inverse pattern, as the amount of UFH in the central compartment increased from 0. These assumptions were used to describe UFH neutralization with excess protamine sulfate and heparin rebound after neutralization.

In Figure 1, *X*_*UFH-C*_ and *X*_*UFH-P*_ are represent the amounts of UFH in central and peripheral compartments, whereas *V*_*UFH-C*_ and *V*_*UFH-P*_ and are the apparent volumes of distribution for the central and peripheral compartments, respectively; *K*_*10*_ is the first-order elimination rate constant; *K*_*12*_ and *K*_*21*_ and are the first-order rate constants of the transportations of UFH from the central to the peripheral compartment and from the peripheral to the central compartment, respectively.

The original differential equations corresponding to the two-compartment model with single intravenous administration and first-order elimination are given in *Eq.*1 and *Eq.*2.

1 $$ \frac{d{X}_{UFH-C}}{dt}={K}_{21}{X}_{UFH-P}-{K}_{12}{X}_{UFH-C}-{K}_{10}{X}_{UFH-C} $$

2 $$ \frac{d{X}_{UFH-P}}{dt}=-{K}_{21}{X}_{UFH-P}+{K}_{12}{X}_{UFH-C} $$

The initial condition: *t* = 0, *X*_*UFH* − *C*_ = *D*_*inj*_, *X*_*UFH* − *P*_ = 0, where *t* is time, and *D*_*inj*_ is the injected dose of UFH. To be more intuitive, we excluded the algebraic steps, and the Laplace-transformed equations of integral form are given by *Eq.*3, 4, 5, 6.

3 $$ \alpha =\frac{\left(\left({K}_{10}+{K}_{12}+{K}_{21}\right)+\sqrt{{\left({K}_{10}+{K}_{12}+{K}_{21}\right)}^2-4{K}_{10}{K}_{21}}\right)}{2} $$

4 $$ \beta =\frac{\left(\left({K}_{10}+{K}_{12}+{K}_{21}\right)-\sqrt{{\left({K}_{10}+{K}_{12}+{K}_{21}\right)}^2-4{K}_{10}{K}_{21}}\right)}{2} $$

5 $$ {X}_{UFH-C}={f}_1(t)=\frac{\left(\alpha -{K}_{21}\right){D}_{inj}{e}^{-\alpha t}+\left({K}_{21}-\beta \right){D}_{inj}{e}^{-\beta t}}{\alpha -\beta } $$

6 $$ {X}_{UFH-P}={f}_2(t)=\frac{-{K}_{12}{D}_{inj}{e}^{-\alpha t}+{K}_{12}{D}_{inj}{e}^{-\beta t}}{\alpha -\beta } $$

Thus, we have the following equation representing the time-sequence function of the plasma UFH level, shown in *Eq.*7:

7 $$ {C}_{UFH-C}={f}_3(t)=\frac{\left(\alpha -{K}_{21}\right){D}_{inj}{e}^{-\alpha t}+\left({K}_{21}-\beta \right){D}_{inj}{e}^{-\beta t}}{\left(\alpha -\beta \right)\left(V/F\right)} $$

Irregular intra-venous injection of UFH could be administered when there was a risk of blood coagulation during CPB. Thus, the equations representing the model of a single intravenous injection of UFH (*Eq.*5, 6, 7) were updated for multiple irregular intravenous injections of UFH, which is given by *Eq.*8–10 a according to the “superposition principle” of linear systems.

8 $$ {X}_{UFH-C}={g}_1(t)={\displaystyle \sum_{i=1}^n}\frac{\left(\alpha -{K}_{21}\right){D}_i{e}^{-\alpha \left(t-{\tau}_i\right)}+\left({K}_{21}-\beta \right){D}_i{e}^{-\beta \left(t-{\tau}_i\right)}}{\alpha -\beta } $$

9 $$ {X}_{UFH-C}={g}_1(t)={\displaystyle \sum_{i=1}^n}\frac{\left(\alpha -{K}_{21}\right){D}_i{e}^{-\alpha \left(t-{\tau}_i\right)}+\left({K}_{21}-\beta \right){D}_i{e}^{-\beta \left(t-{\tau}_i\right)}}{\alpha -\beta } $$

10 $$ {C}_{UFH-C}={g}_3(t)={\displaystyle \sum_{i=1}^n}\frac{\left(\alpha -{K}_{21}\right){D}_i{e}^{-\alpha \left(t-{\tau}_i\right)}+\left({K}_{21}-\beta \right){D}_i{e}^{-\beta \left(t-{\tau}_i\right)}}{\left(\alpha -\beta \right)\left(V/F\right)} $$

Where *τ*_*i*_ is the time of the *i*^*th*^ administration of UFH; *D*_*i*_ is the dose of the *i*^*th*^ administration of UFH; and the maximum number of UFH doses is counted as *n*.

The amount of UFH in the central compartment must clear instantly when protamine sulfate is administered for neutralization. To describe such a situation after neutralization or the post-CPB pharmacokinetics of UFH mathematically, the initial conditions of the differential equations (*Eq.*1–2) mentioned above were reset to *t* = *T*_*neu*_, *X*_*UFH* − *C*_ = 0, *X*_*UFH* − *P*_ = *g*_2_(*T*_*neu*_), where *T*_*neu*_ represents the time of neutralization using protamine sulfate, and the function *g*_2_ represents Eq. 9. Then *Eq.*5–7, representing the amounts of UFH in the central and peripheral compartments as well as the plasma UFH level, were updated under new initial conditions, resulting in *Eq.*11, 12, 13:

11 $$ {X}_{UFH-C}={h}_1(t)=\frac{g_2\left({T}_{neu}\right)\left(\alpha -{K}_{21}\right)\left(\beta -{K}_{21}\right)\left[{e}^{\left(\beta -{K}_{12}-{K}_{21}-{K}_{10}\right)\left(t-{T}_{neu}\right)}-{e}^{\left(\alpha -{K}_{12}-{K}_{21}-{K}_{10}\right)\left(t-{T}_{neu}\right)}\right]}{K_{12}\left(\alpha -\beta \right)} $$

12 $$ {X}_{UFH-P}={h}_2(t)=\frac{g_2\left({T}_{neu}\right)\left[\left(\alpha -{K}_{21}\right){e}^{\left(\alpha -{K}_{12}-{K}_{21}-{K}_{10}\right)\left(t-{T}_{neu}\right)}-\left(\beta -{K}_{21}\right){e}^{\left(\beta -{K}_{12}-{K}_{21}-{K}_{10}\right)\left(t-{T}_{neu}\right)}\right]}{\alpha -\beta } $$

13 $$ {C}_{UFH-C}={h}_3(t)=\frac{g_2\left({T}_{neu}\right)\left(\alpha -{K}_{21}\right)\left(\beta -{K}_{21}\right)\left[{e}^{\left(\beta -{K}_{12}-{K}_{21}-{K}_{10}\right)\left(t-{T}_{neu}\right)}-{e}^{\left(\alpha -{K}_{12}-{K}_{21}-{K}_{10}\right)\left(t-{T}_{neu}\right)}\right]}{K_{12}\left(\alpha -\beta \right)\left(V/F\right)} $$

Thus, the overall time sequence function of plasma UFH during and after CPB should combine *Eq*. 10 and *Eq*. 13, as follows: and together, as described below:

14 $$ {\mathrm{C}}_t=\left\{\begin{array}{c}\hfill {g}_3(t)={\displaystyle \sum_{i=1}^n}\frac{\left(\alpha -{K}_{21}\right){D}_i{e}^{-\alpha \left(t-{\tau}_i\right)}+\left({K}_{21}-\beta \right){D}_i{e}^{-\beta \left(t-{\tau}_i\right)}}{\left(\alpha -\beta \right)\left(V/F\right)} when\ 0\le t<{T}_{neu}\hfill \\ {}\hfill {h}_3(t)=\frac{g_2\left({T}_{neu}\right)\left(\alpha -{K}_{21}\right)\left(\beta -{K}_{21}\right)\left[{e}^{\left(\beta -{K}_{12}-{K}_{21}-{K}_{10}\right)\left(t-{T}_{neu}\right)}-{e}^{\left(\alpha -{K}_{12}-{K}_{21}-{K}_{10}\right)\left(t-{T}_{neu}\right)}\right]}{K_{12}\left(\alpha -\beta \right)\left(V/F\right)}\  when t\ge {T}_{neu}\hfill \end{array}\right. $$

As shown in Eq. 14, the pharmacokinetics of UFH comprised two mathematically different stages. To have a simple iteration step and a short computation time and, most importantly, to set the amount of UFH in the central compartment as 0 at the time of neutralization, the Laplace-transformed equation (*Eq.*14) along with the $PRED block in NONMEM was used in the computation process.

During computation, *h*_1–3_(*t*) and *g*_1–3_(*t*) were calculated simultaneously, and their real-time values documented automatically by NONMEM. At the time of neutralization, the value of *X*_*UFH* − *C*_ was instantly set to 0, whereas the value of *X*_*UFH* − *P*_ at that moment was documented automatically as the new initial condition of the inverse distribution of UFH from the peripheral compartment towards the central compartment.

Population pharmacokinetic modeling involves random effects and fixed effects [15]. In this study, the random effects were interindividual and residual. The inter-individual random effects were analyzed using an exponential model (*Eq.*15), whereas the residual random effects were evaluated using a hybrid model (*Eq.*16):

15 $$ {P}_i={P}_{pop}\cdotp {e}^{\eta_i} $$

Where *P*_*i*_ is the pharmacokinetic parameter of the *i*^*th*^ individual; *P*_*pop*_ is the typical population parameter; *η*_*i*_ is the inter-individual variability of the *i*^*th*^ individual, following a normal distribution of *N*(0, *ω*^2^)

16 $$ \left\{\begin{array}{c}\hfill {C}_{pred}=\frac{X_{UFH-C}}{V_{UFH-C}}\hfill \\ {}\hfill {C}_{obs}={C}_{pred}\cdotp \cdotp \left(1+{\varepsilon}_1\right)+{\varepsilon}_2\hfill \end{array}\right. $$

In *Eq*. 16, *C*_*obs*_ is the observed plasma concentration, C_pred_ is the predicted plasma concentration, and *ε*_1_ and *ε*_2_ define the proportional error and additional error, respectively, following a normal distribution of *N*(0, *σ*_1_^2^) and *N*(0, *σ*_2_^2^).

Continuous fixed effects (age, body weight) were analyzed in the pharmacokinetic model in a linear manner:

17 $$ {P}_i={P}_{pop}\cdotp \cdotp \left(1\pm {\theta}_{COV}\cdotp \cdotp \left(\overline{COV}-CO{V}_i\right)\right)\cdotp \cdotp {e}^{\eta_i} $$

Where *P*_*i*_ and *P*_*pop*_ are the individual and population pharmacokinetic parameters, *θ*_*COV*_ is the influence coefficient of the given fixed effect, and $\overline{COV}$ and $\underset{\bar{\mkern6mu}}{CO{V}_i}$ are the mean and individual values of the fixed effect.

Discontinuous fixed effects (e.g. sex) were analyzed in the pharmacokinetic model in a conditional manner:

18 $$ {P}_i={P}_{pop}\cdotp {e}^{\eta_i}\cdotp {\theta}^{GNDR} $$

Where *GNDR* is the value of represents sex (0 for male, 1 for female). *θ* is the influence coefficient of sex. *θ*^*GNDR*^ equals 1 for males and *θ* when it is for females.

Covariate analysis was performed by a stepwise regression known as “forward inclusion” and “backward elimination”. In forward modeling, all covariates were added to the base model, one after the other. Then, all covariates with a decrease in the objective function value (OFV) over 6.63 (*x*^2^ distribution with *1df* for p < 0.01) were listed in descending order according to the decrease in OFV. All remaining covariates were again added to the base model in order. If the OFV reduction was over 6.63 (p < 0.01), the covariate was retained. Otherwise, it was ruled out until no further reduction of the OFV occurred (full model).

In backward modeling, all covariates in the full model were removed one at a time. Only covariates with sufficient contributions to the prediction of the pharmacokinetic model were retained based on an increase in the OFV greater than 10.83 (p < 0.001). Otherwise, the covariate was ruled out until no further increase of the OFV occurred (final model).

### Model validation

The basic goodness-of-fit plots, including population predicted concentration (PRED) *vs*. observed concentration (OBS) plot, individual predicted concentration (IPRED) *vs*. OBS plot, conditional weighted residuals (CWRES) *vs*. PRED plot, and CWRES *vs*. time plot, were used to evaluate the final model.

The bootstrap method was used to evaluate accuracy and stability. The original dataset was re-sampled randomly 1000 times, producing 1000 new datasets. The final model was recalculated for the new datasets, and the median value and 90% confidence intervals of the recalculated model parameters compared to the final model.

The visual predictive check (VPC) method was used to evaluate the accuracy and predictive ability of the final model. The final model with the original dataset was simulated 1000 times with different random seeds, and the 5%, 50%, and 95% fractiles along with the 90% confidence interval of 1000-fold simulation compared to the observed data.

We also simulated the administration of excess protamine via bolus injection or infusions to determine which provides minimal heparin rebound after CPB. Simulations were performed with NONMEM and the results compared to identify the optimal treatment for heparin rebound.

## Results

**Figure 2:**
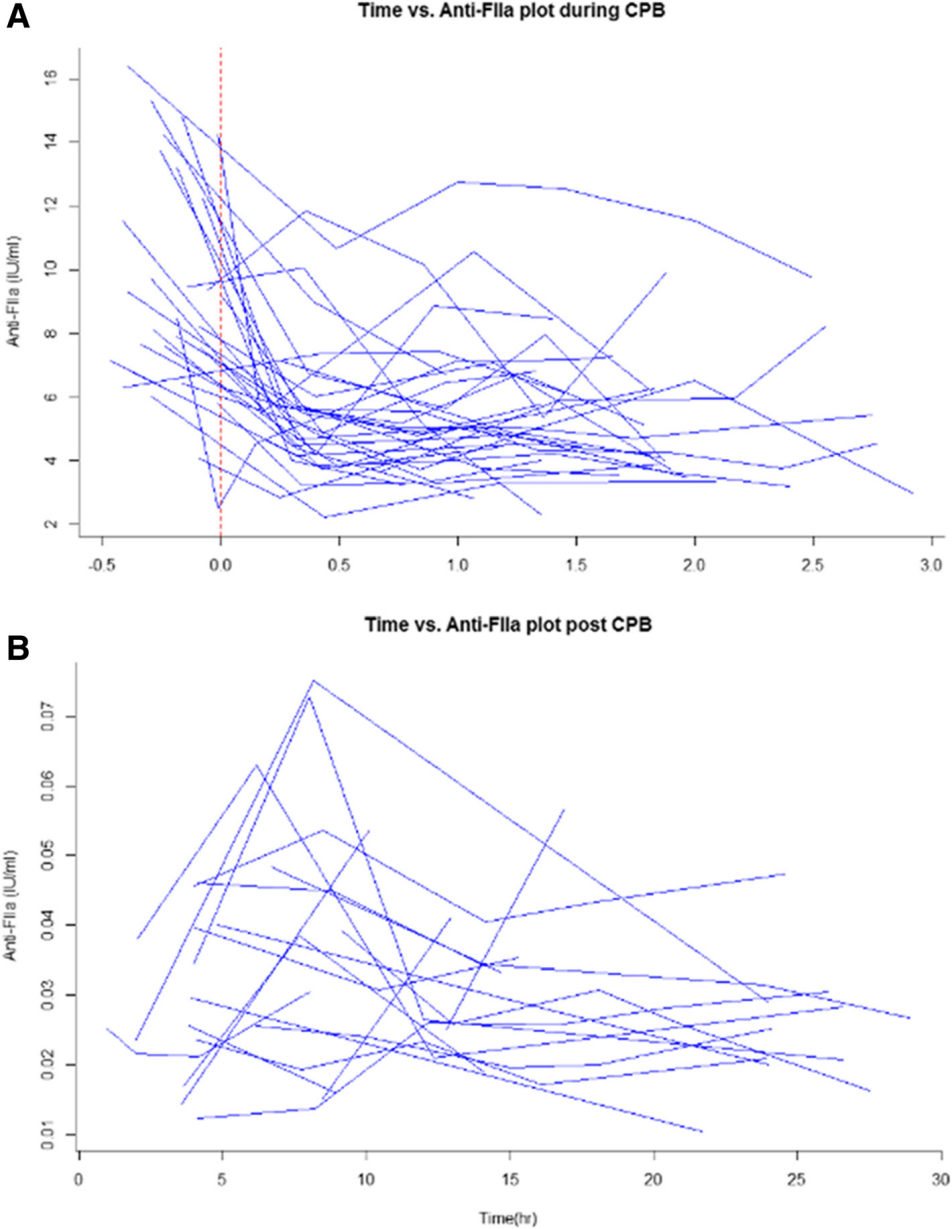
**Anti-F**_**IIa**_ **activity** ***vs*** **.** Time. **A**, Anti-F_IIa_ activity *vs*. time for blood samples obtained during CPB. **B**, anti-F_IIa_ activity *vs*. time for blood samples obtained no less than24 h after the end of CPB (n = 32). In both plots, the time at the start of CPB was set to 0.

Thirty-two patients completed the clinical study. Total administered UFH dose was about 33000 IU/surgery. The change in anti-F_IIa_ during CPB and 24 h after the end of CPB is shown in Figure 2.

**Figure 3:**
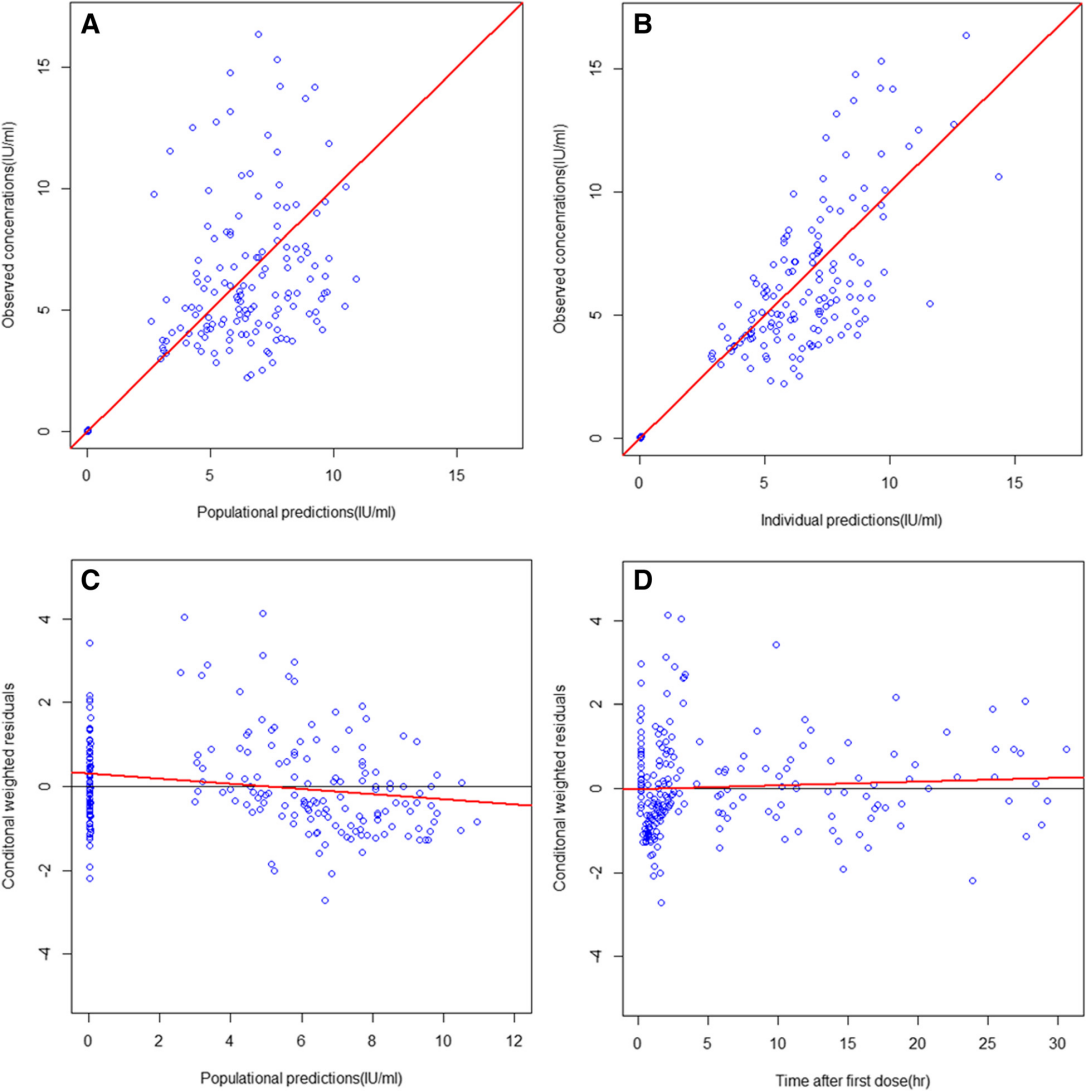
**Basic goodness-of-fit plots of the final population pharmacokinetic model. A**: observed *vs*. population predicted concentrations. **B**: observed *vs*. individual predicted concentrations. **C**: conditional weighted residuals *vs*. population predicted concentrations. **D**: conditional weighted residuals *vs*. time after first dose. The red line represents the linear fit by the ordinary least square (OLS) method.

The model was parameterized in terms of volume of distribution and clearance rather than rate constants. The inter-individual random effect was evaluated with an exponential error model and the intra-individual random effect was evaluated with a hybrid model, involving additive and proportional errors. The fixed effects of covariates were tested (age, body weight, sex) with forward modeling and backward elimination methods. None of the tested covariates significantly decreased the objective function, and thus did not improve the fit. The goodness-of-fit plots of the final model are shown in Figure 3; estimates of the pharmacokinetic parameters of the final model along with the results of Bootstrap analyses are listed in Table 2.

**Table 2:** **Estimates of the parameters of the final model and results of Bootstrap analyses**

**Parameter**	**Final model estimate (RSE%)**	**Bootstrap median**	**Bootstrap CI (95%)**
CL_UFH, 1-order_ (L·h^–1^)	1.18 (7.25)	1.18	0.99, 1.35
V_UFH-C_ (L)	3.04 (8.09)	3.02	2.57, 3.51
Q_UFH_ (L·h^–1^)	0.171 (14.40)	0.160	0.0977, 0.311
V_UFH-P_ (L)	8.01 (31.8)	7.01	2.63, 27.6
ω^2^_CL(UFH, 1-order)_	0.122 (77.7)	0.126	0.00452, 0.310
ω^2^_V(UFH-C)_	0.105 (48.7)	0.0966	0.0239, 0.197
ω^2^_Q(UFH)_	0.0978 (76.9)	0.105	0.00498, 0.259
ω^2^_V(UFH-P)_	0 (FIX)	0 (FIX)	0 (FIX)
σ^2^_pro(UFH)_	0.139 (14.2)	0.135	0.105, 0.167
σ^2^_add(UFH)_	0 (FIX)	0 (FIX)	0 (FIX)

**Figure 4:**
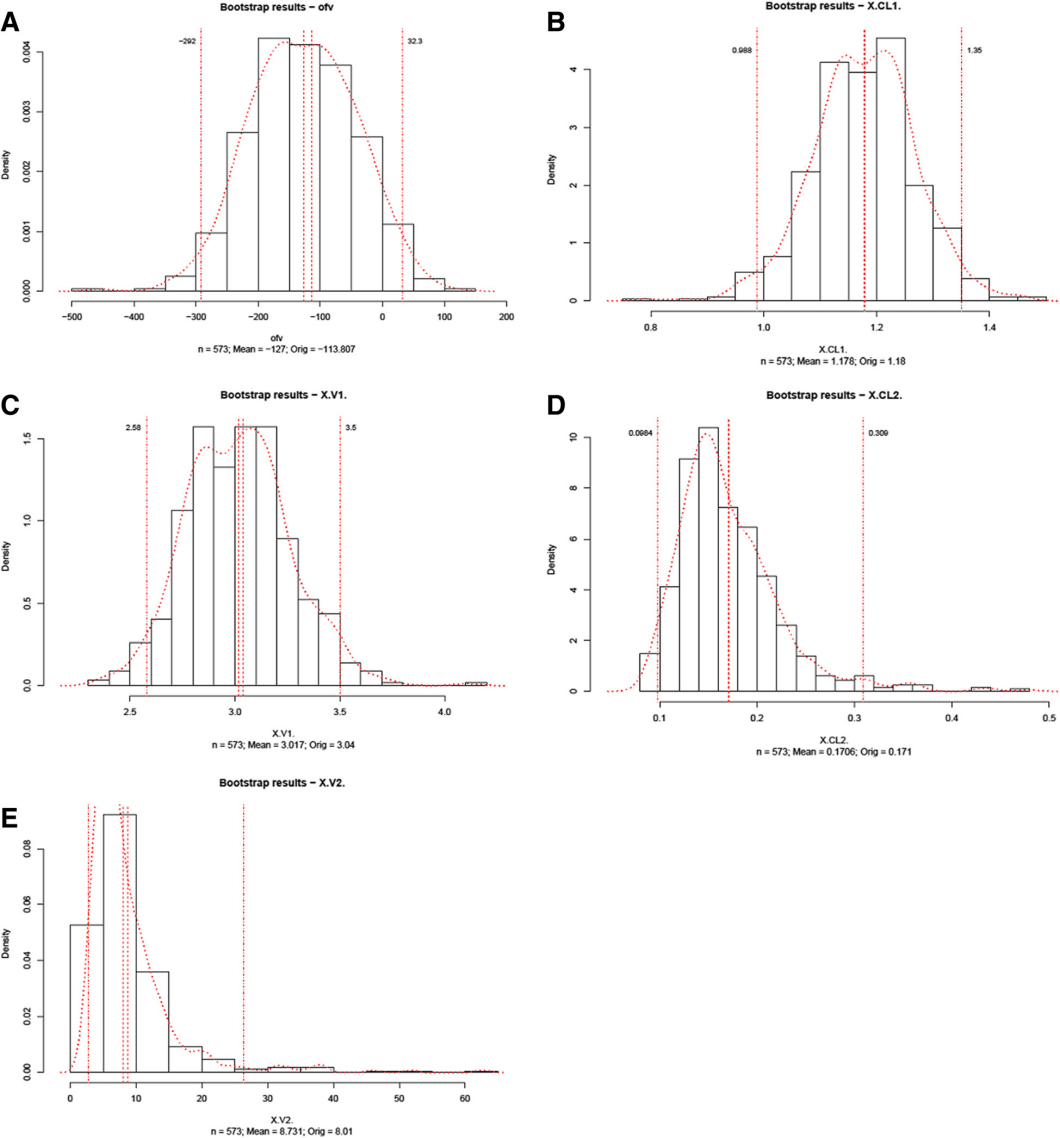
**Distributions of the OFV and key parameters in bootstrap analyses. A**: OFV; **B**: CL/F; **C**: V_UFH-C_ in Bootstrap analyses; **D**: Q/F; **E**: V_UFH-P_.

**Figure 5:**
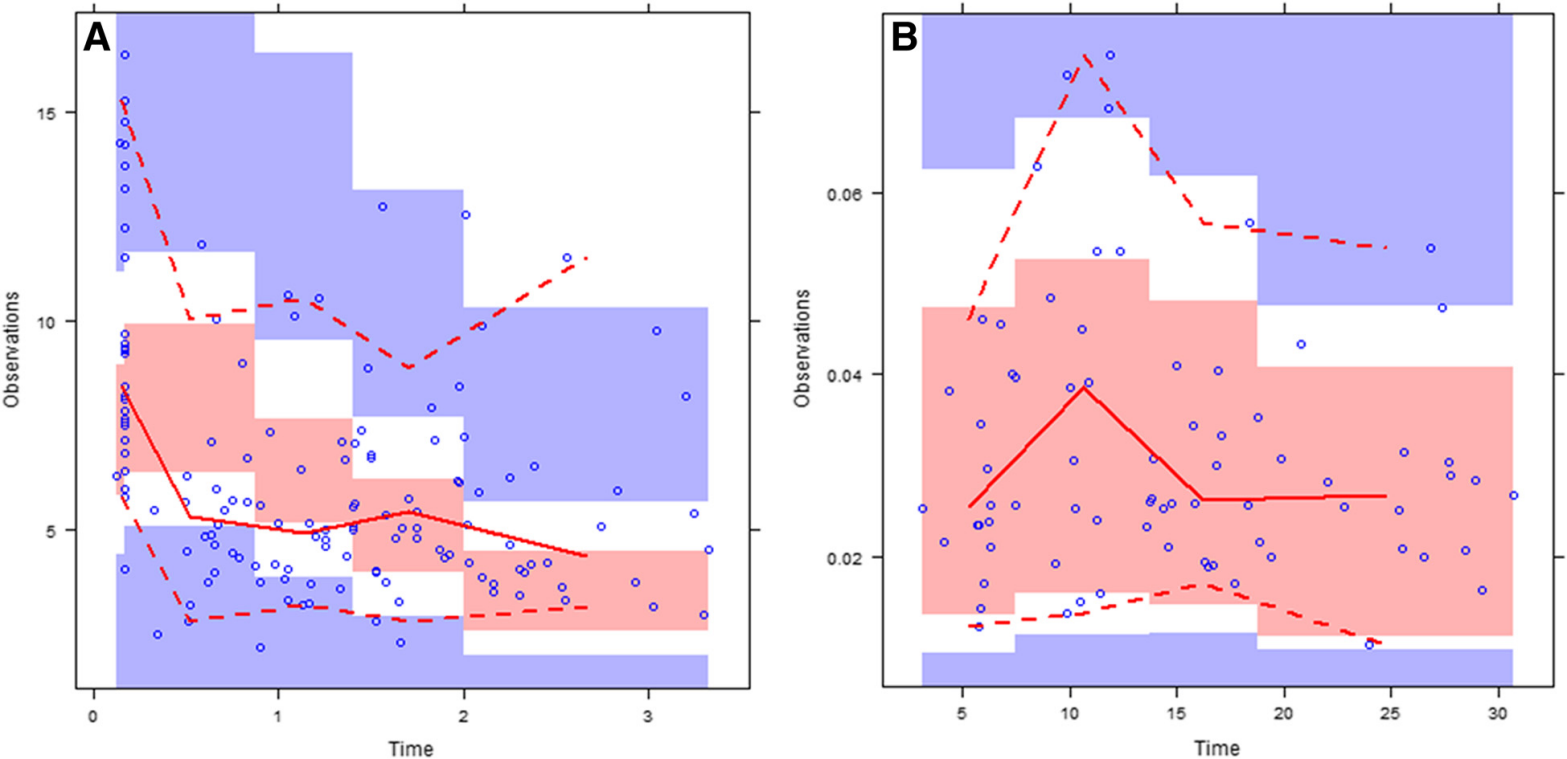
**VPC plots of the final model. A**: before protamine neutralization; **B**: post-CPB VPC. Blue-colored areas are the 90% CI and pink-colored areas are the 50% CI for simulated concentrations. The red line represents the median of the observed concentration; the dashed lines represent the upper and lower 90% CI of observed concentrations.

**Figure 6:**
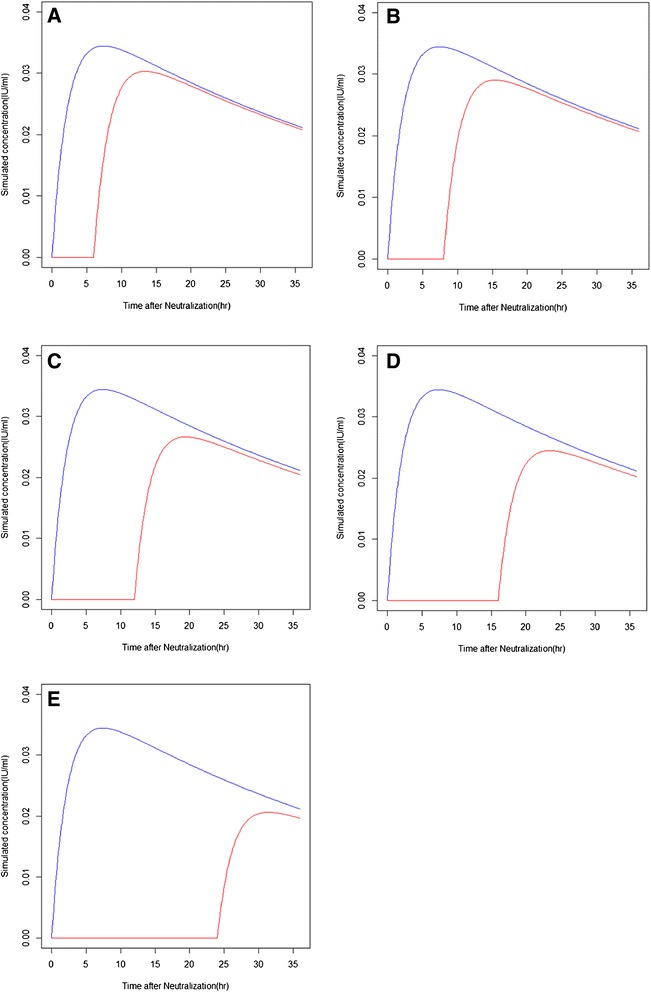
**Simulated plots of infusion times and a protamine infusion rate of 25 mg/h. A**: 6-h infusion; **B**: 8-h infusion; **C**: 12-h infusion; **D**: 16-h infusion; **E**: 24-h infusion. Blue lines are simulated concentrations of UFH without follow-up protamine infusions or doses. Red lines denote simulated concentrations of UFH over different protamine infusion times.

The final model was validated with Bootstrap and VPC. Bootstrap analyses showed a success rate of 57.3% (573 out of 1000 were successful in covariance steps, whereas 982 out of 1000 were successful in minimization). Parameter distribution in Bootstrap analyses is summarized in Figure 4 and the VPC result shown in Figure 5. Simulated plasma Anti-F_IIa_ activities of a hypothetical individual whose pharmacokinetic parameters were identical to the parameter estimations in our final model are shown in Figure 6.

## Discussion

The results suggested that UFH (375 IU/kg) administered during CPB follows a two-compartment distribution and first-order elimination curve with an approximate initial half-life of 90 min. Median plateau anti-F_IIa_ activity during CPB was 2–19 IU/mL (Figure 5), which was within the therapeutic range of UFH during CPB [16]. Thus, UFH levels in CPB may exhibit very large interindividual differences. The median anti-F_IIa_ level at the end of CPB was 4.8 IU/mL and was neutralized with a mean 2.04 h after the start of CPB. A heparin-rebound peak of 0.04 IU/mL was attained 8 h after the end of CPB and was maintained above 0.02 IU/mL 24 h after neutralization, evidence of heparin rebound.

Based on patient plasma UFH levels, a population pharmacokinetic model was established using NONMEM. According to the goodness-of-fit plots in Figure 3, CWRES seems to be distributed randomly between −4 to +4 during and after CPB, and the population predictions *vs*. observations were distributed along the y = x line, suggesting that our final model was not biased and was consistent.

Continuous infusion of protamine (25 mg/h) after CPB can reduce the severity of post-operative heparin rebound [4]. Thus, for a better illustration of our final model and to test its clinical utility, we simulated the plasma Anti-F_IIa_ activities of a hypothetical individual whose pharmacokinetic parameters were identical to the parameter estimations in our final model. That is, under hypothetical situations, continuous protamine infusions at 25 mg/h lasting 6–24 h were administered after CPB. The result of this simulation was similar to prior reports (Figure 6) [4]. A comparison of plots A and E suggest a 6-h infusion would decrease the plasma heparin levels caused by heparin rebound. A 24-h infusion would provide even better control.

Most studies on UFH pharmacokinetics have been based on measuring anti-coagulation activity as represented by the activated partial thromboplastin time or activated coagulation time in plasma or whole blood. Such methods are unstable, have poor reproducibility across assays and, most importantly, can be used only to monitor plasma UFH levels during or after CPB [17-20]. In our study, patient plasma levels of UFH were monitored during and after CPB using an established anti-F_IIa_ assay [21,22]. Heparin rebound could be caused by the inverse distribution of UFH from the peripheral compartment to the central compartment. Therefore, additional administration of protamine after CPB could reduce the intensity and duration of post-CPB heparin rebound.

To our knowledge, this is the first report of a UFH pharmacokinetic model for CPB surgery; the model can predict UFH concentrations at the end of CPB to guide optimal neutralization with protamine sulfate. Study limitations include the fact that only anti-F_IIa_ activity was monitored and UFH metabolites were not studied, which means the roles of LMWH in heparin rebound and during CPB remain unknown. In addition, the study patients were given several concomitant drugs that may also have affected coagulation.

## Conclusions

A two-compartment model demonstrates the precise pharmacokinetics of UFH in Chinese patients during CPB and explains the postoperative heparin rebound after protamine neutralization. A 24-h protamine infusion is more effective than the 6-h infusion method for reducing plasma heparin levels caused by heparin rebound.
